# Supplementary figures and images for: Prevalence and factors associated with acute respiratory infection among under-five children in selected tertiary hospitals of Kathmandu Valley
Source: PLoS One. 2022 Apr 7;17(4):e0265933. doi: 10.1371/journal.pone.0265933 (PMC8989212; doi:10.1371/journal.pone.0265933)

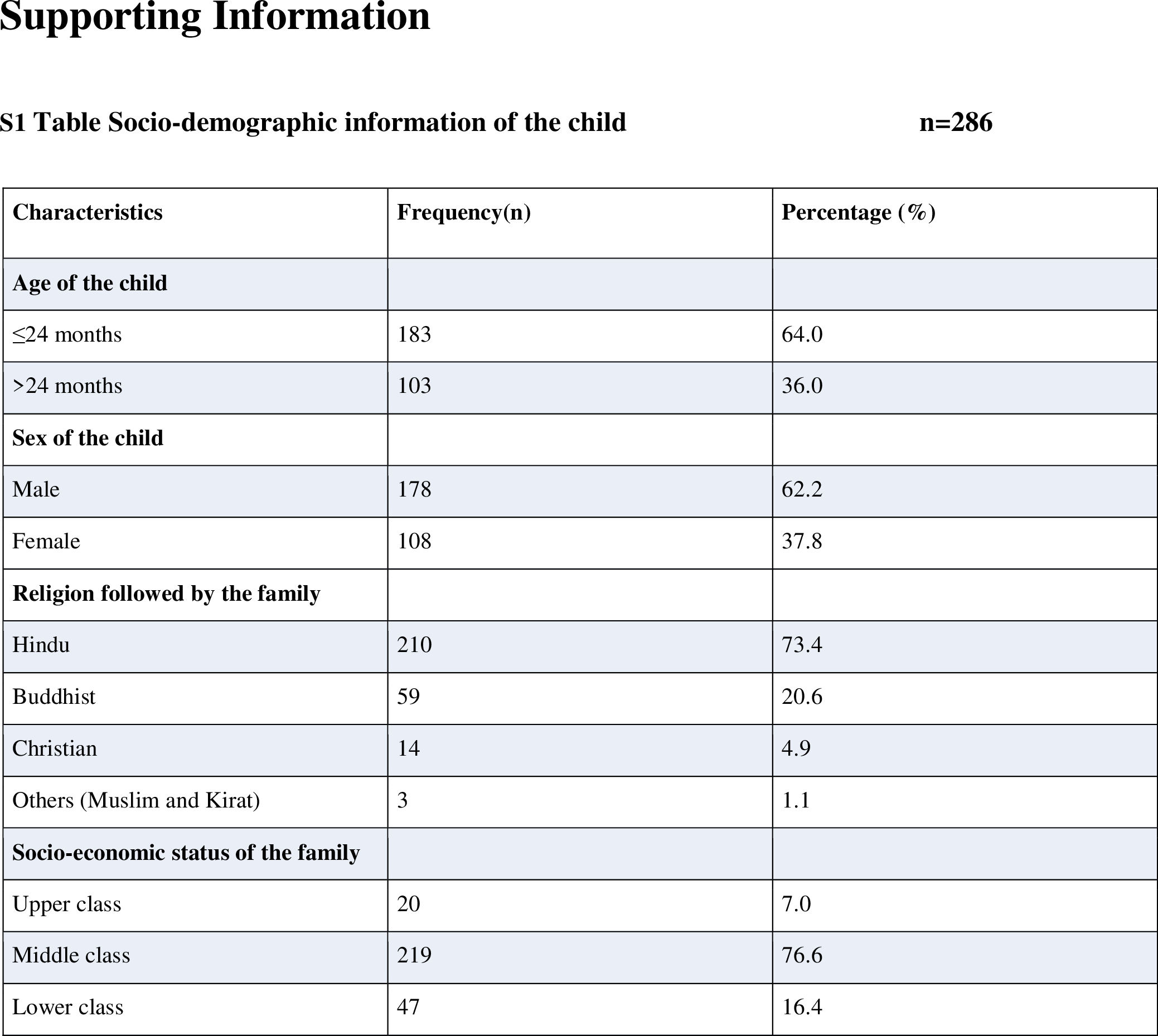

Supplement: S1 Table — n = 286. (TIF) [file pone.0265933.s001.tif]

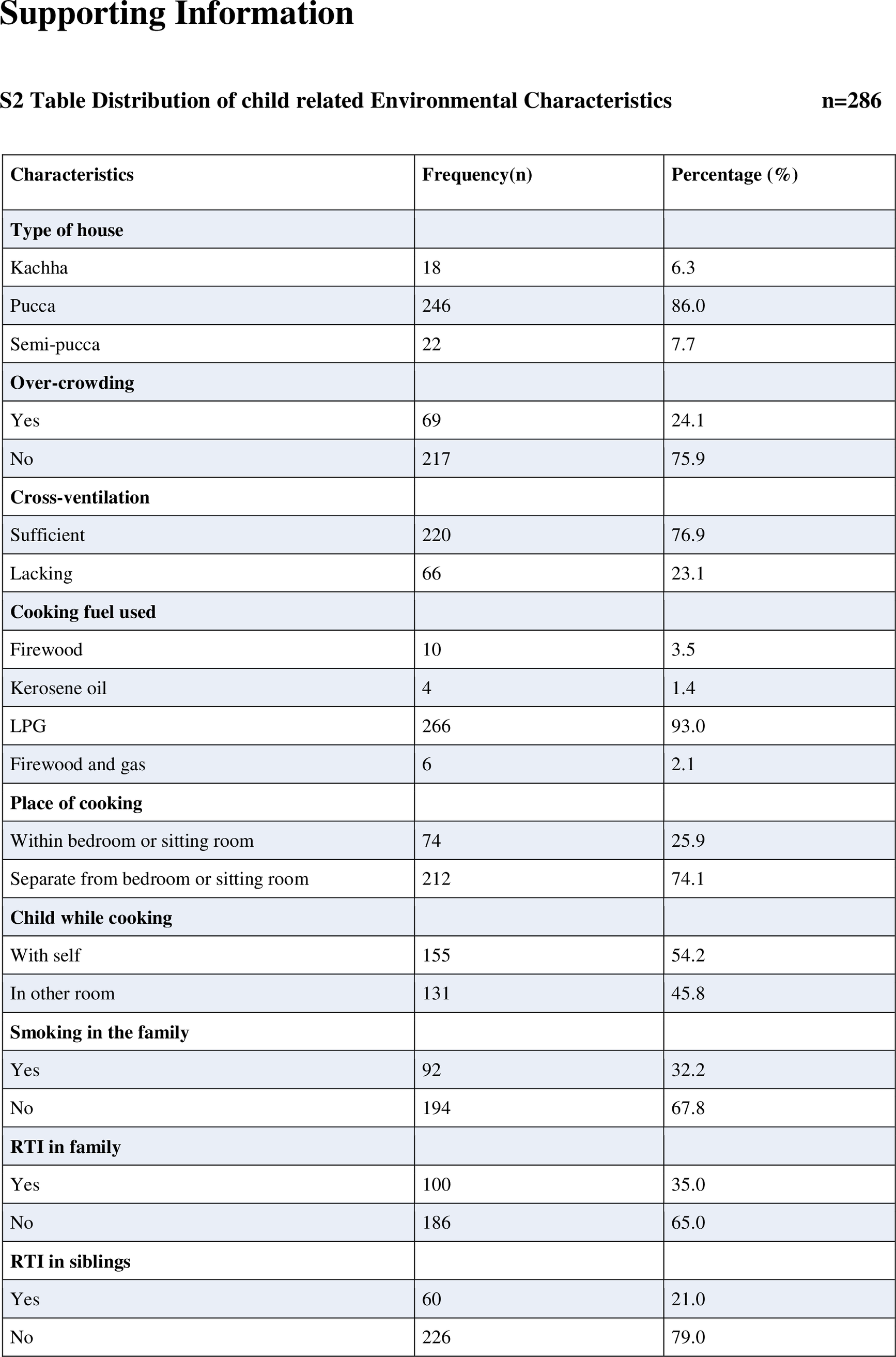

Supplement: S2 Table — n = 286. (TIF) [file pone.0265933.s002.tif]

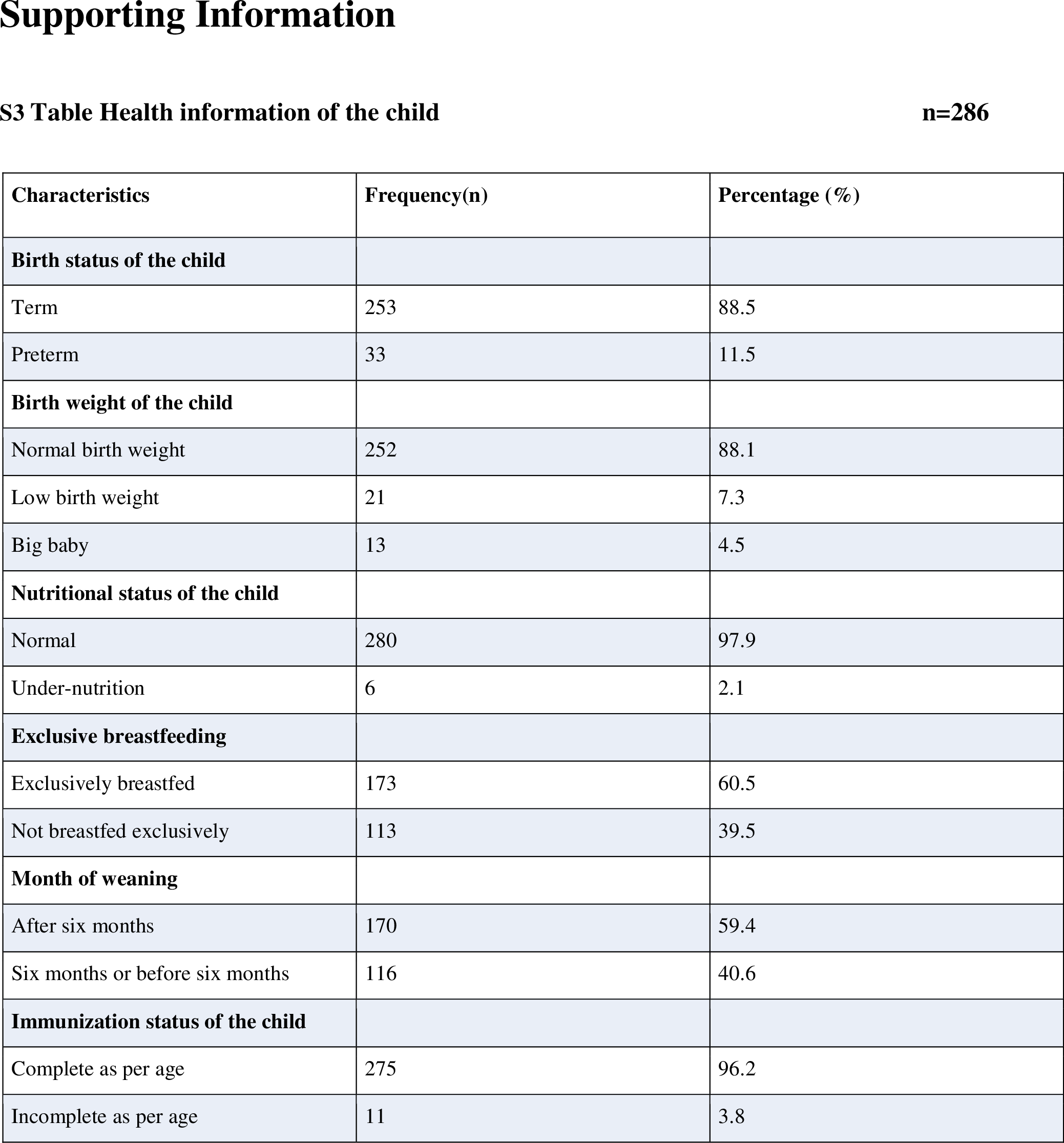

Supplement: S3 Table — n = 286. (TIF) [file pone.0265933.s003.tif]
