# Supplementary material for: Prevalence and factors associated with acute respiratory infection among under-five children in selected tertiary hospitals of Kathmandu Valley
Source: PLoS One. 2022 Apr 7;17(4):e0265933. doi: 10.1371/journal.pone.0265933 (PMC8989212; doi:10.1371/journal.pone.0265933)
Supplement: Attachment — Submitted filename: Response to Reviewers.docx [file pone.0265933.s004.docx]

Dear reviewers,

We thank for your critical assessment of our work. We have revised and edited our manuscript and in the following we address your concerns point by point.

We would please to hear your recommendations for further improvement of this manuscript.

**Response to Academic editor**

1. Please ensure that your manuscript meets PLOS ONE's style requirements, including those for file naming.

Author Response: The manuscript has been revised and corrected as per the requirements of PLOS ONE.

2. In your Data Availability statement, you have not specified where the minimal data set underlying the results described in your manuscript can be found.

Author Response: All the data are with in the manuscript and under supporting information file.

**Response to the reviewer’s comment**

1. Comment 3: Have the authors made all data underlying the findings in their manuscript fully available?

Author Response: All the data are made available within the manuscript except the one that are inside the supporting information file.

2. Comment 4: Is the manuscript presented in an intelligible fashion and written in standard English?

Author Response: The manuscript has been revised, and corrected based on the standard format.

3. Comment 5: Reviewer #1

- The manuscript is not presented in an intelligible fashion and is written in standard English. Authors need to read this paper carefully and make all the necessary changes, as there are several errors while reporting this paper.

Author Response: The manuscript has been revised and corrected making all the necessary changes.

4. Comment 5: Reviewer #2

- Abstract: A few clarifications needed. Please reword:
- Acute respiratory infection (ARI) is responsible for 30-50 percent of visits to health facility and 20-30 percent of admissions to hospital in Nepal. – is this for both adults and children or for children under 5 years old?

Author Response: The data is for under-five children.

- What does it mean, ‘incidence of ARI/1000 children < 5 years of age is 344 in Nepal’? Does this mean 344/1000? Please reword: The incidence of ARI in children < 5 years old is 344/1000. The sentences are not clear. Please reword to make it clear.

Author Response: The sentence has been reworded to “Incidence of ARI among under-5 years of age is 344 per 1000 children in Nepal.”

- Non-probability is not equivalent to purposive. Purposive is to target those with ARI. In this study, I believe it is convenient sampling – please stick to convenient sampling.

Author Response: Changed to convenient sampling.

- When you say out of the 286 children, half had ARI. What did the other children have?

Author Response: It means, out of the total participants, half of them had ARI while others were visiting OPD for other cause.

- Also, do not include conclusion that male children are not susceptible to ARI, because the results were insignificant.

Author Response: The sentence has been omitted.

- Introduction
- Line 60: ARI is responsible for about 30-50% of visits…. Question: is this in children under 5 years old? Please specify.

Author Response: The data is for under-five children. The sentence has been reworded.

- Line 64: The deaths due to ARI at health facility is 127. What does this mean? Any health facility or hospitals or primary care? Can remove this information as it is not clear.

Author Response: The sentence has been removed.

- Methodology
- Line 91: Please specify the time and duration of the data collection period e.g. from month, year until month, year.

Author Response: The duration for data collection has been specified.

- Line 106: Please add the Cronbach’s alpha for the pretesting that you have done, to show whether the questionnaire was reliable.

Author Response: The calculated value of Cronbach’s alpha was 0.85, which is mentioned in the “Instruments” section under methods.

- From paragraph 66-73 in the introduction, please add in the methodology the factors that was studied in your study.

Author Response: It has been mentioned as “variables under study” under methods.

- Chi-squared test was done to show relationship between the categorical variables. My personal opinion is it is better to do simple logistic regression, and then move on to multiple logistic regression for the significant factors. Any reason why you use Chi-squared test rather than simple logistic?

Author Response: Simple logistic regression has been carried for bivariate analysis.

- Discussion
- 218: In the discussion, you do not need to mention twice but to just discuss about the three factors that were associated with increase risk of ARI in children, which are religion, presence of child in the kitchen while cooking and presence of respiratory tract infection in family member.

Author Response: The factors has been discussed once.
